# Two new toxic yellow *Inocybe* species from China: morphological characteristics, phylogenetic analyses and toxin detection

**DOI:** 10.3897/mycokeys.81.68485

**Published:** 2021-08-03

**Authors:** Sai Nan Li, Fei Xu, Ming Jiang, Feng Liu, Fang Wu, Ping Zhang, Yu Guang Fan, Zuo Hong Chen

**Affiliations:** 1 College of Life Sciences, Hunan Normal University, Changsha, Hunan 410006, China Hunan Normal University Changsha China; 2 Ningxia Center for Disease Control and Prevention, Yinchuan, Ningxia 750004, China Ningxia Center for Disease Control and Prevention Yinchuan China; 3 Hunan Edible Fungal Research Institute, Changsha, Hunan 410013, China College of Life Science, Hunan Normal University Changsha China; 4 Key Laboratory of Tropical Translational Medicine of Ministry of Education, Hainan Medical University, Haikou, Hainan 571199, China Hainan Medical University Haikou China

**Keywords:** Inocybaceae, muscarine, taxonomy

## Abstract

Some species of *Inocybes. str.* caused neurotoxic poisoning after consumption around the world. However, there are a large number of species in this genus that have not been studied for their toxicity or toxin content. In this study, we report two new toxic yellow *Inocybes. str.* species from China based on morphological characteristics, phylogenetic analyses and toxin detection. Among the two species, *Inocybesquarrosolutea* is reported as a newly recorded species of China. We also describe a new species, *I.squarrosofulva*, which is morphologically similar to *I.squarrosolutea*. The new species is characterized by its ochraceous squarrose pileus, distinctly annulate cortina on the stipe, nodulose basidiospores and thick-walled pleurocystidia. Muscarine in the fruitbodies was detected by UPLC–MS/MS, the content in *I.squarrosolutea* and *I.squarrosofulva* were 136.4 ± 25.4 to 1683.0 ± 313 mg/kg dry weight and 31.2 ± 5.8 to 101.8 ± 18.9 mg/kg dry weight, respectively.

## Introduction

The genus *Inocybe* (Fr.) Fr. was established as a “tribe” of *Agaricus* (Fries 1821) and treated as a genus in 1863 (Fries 1863). Recent studies elevated it to the family rank, known as the Inocybaceae (Matheny 2005, 2009; Matheny et al. 2020). The present Inocybaceae (*Inocybe* sensu lato) is composed of seven genera, namely *Auritella*, *Inosperma*, *Mallocybe*, *Nothocybe*, *Pseudosperma*, *Tubariomyces*, and *Inocybe* sensu stricto. *Inocybes. str.* with about 850 species, turns out to be the largest genus (Matheny et al. 2020), and novel species have continued to be discovered in recent years (Bandini et al. 2020a, 2020b; Fan and Bau 2020; Caiafa 2021; Mešić et al. 2021). Studies on Inocybaceae in China started in the 20^th^ century. From Deng (1963) first reported 15 species of *Inocybes. l.* Until 2020 about 140 species of Inocybaceae had been reported, of which about 120 belong to *Inocybes. str.* (Fan and Bau 2010, 2013, 2014a, 2014b, 2017, 2018, 2020; Fan et al. 2013, 2018). Wang (1979) described a new species, *I.flavobrunnea*, which was the first new species of *Inocybes. str.* in China. After that, Fan et al. have described seven new species of *Inocybes. str.* in China from 2013 to 2020 (Fan and Bau 2013, 2014a, 2014b, 2020; Fan et al. 2018).

Autonomic toxicity disorder, caused by the ingestion of *Inocybes. l.* spp., is an important type of neurotoxic mushroom poisoning. Muscarine is the principal toxin in *Inocybes. l.* (Chen et al. 2016; White et al. 2018). Based on a review of the literature and their own work on toxin detection, Kosentka et al. (2013) reported whether or not muscarine is present in 98 species of Inocybaceae from 1960 to 2013, including 73 species of *Inocybes. str.* Of these 73 taxa, 57 have been reported to contain muscarine. In China, about 21 species of *Inocybes. str.* are considered poisonous (Mao 2006; Bau et al. 2014; Xu et al. 2020). However, only three species (*I.asterospora*, I.aff.ericetorum, *I.serotina*) of *Inocybes. str.*, causing typically muscarinic poisoning incidents, could be identified as containing muscarine (Chen et al. 1987; Xu et al. 2020; Li et al 2021). Among them, *I.asterospora* and I.aff.ericetorum are new toxic *Inocybe* species reported in China. In summary, toxins have only been reported for 75 species of *Inocybes. str.*, and ca. 7% (59 of 850) have been identified as muscarine-containing poisonous mushrooms. Hence, the toxicity of a large number of *Inocybes. str.* species is still unknown.

In this study, we 1) report *I.squarrosolutea* as a newly recorded species of China, and redescribed this species based on Chinese materials; 2) describe a new species of *Inocybes. str.*, based on morphological and phylogenetic evidence; and 3) characterize the muscarine content of these two species by UPLC–MS/MS.

## Materials and methods

### Specimen collection and drying treatment

Most other specimens were collected from Hunan Province; only one specimen was collected from Huang Mountain, Anhui Province. The fresh basidiomata were dried using an electric dryer EVERMAT operated at 45 °C for 10 h. The dried specimens, along with the holotype of the newly described species, were deposited in the Mycological Herbarium of Hunan Normal University (**MHHNU**), Changsha, China. A small piece of fresh basidioma was also dried with silica gel for molecular analysis.

### Morphological studies

Specimens were photographed in situ using a Sony digital camera (LICE-7, Sony, Tokyo, Japan). The macromorphological characters of fresh mushrooms were recorded as soon as possible after collection. Color codes were described following Kornerup and Wanscher (1978). Microscopic structures were studied from dried materials mounted in 5% aqueous KOH, and Congo red was used as a stain when necessary. All the measurements were performed at 1000× magnification, and a minimum of 20–30 basidiospores from each basidioma were measured in side view. Micromorphological investigations were performed by means of a Nikon Eclipse 50i microscope (Nikon, Tokyo, Japan). The measurement methods followed those of Fan et al. (2013). Dimensions of basidiospores and Q values were given as (a) b–c (d), where “b–c” cover a minimum of 90% of the measured values, and “a” and “d” represent extreme values; Q is the ratio of length to width in an individual basidiospore. Qm is the average Q of all basidiospores ± sample standard deviation. The descriptive terms are in accordance with Fan and Bau (2020), Horak et al. (2015), and Matheny et al. (2012). SEM images of basidiospores were obtained using a scanning electron microscope JSM-6380LV (JEOL Ltd., Tokyo, Japan).

### DNA extraction, amplification, and sequencing

DNA was extracted from dried basidiomata using a fungal DNA extraction kit manufactured by Omega Bio-Tek (Norcross, GA, USA). The following primer pairs were used for PCR amplification and sequencing: ITS5 and ITS4 for the internal transcribed spacer (ITS) region (White et al. 1990); LR0R and LR5 for the nuclear ribosomal large subunit (nrLSU) region (Vilgalys and Hester 1990); and bRPB2-6F and bRPB2-7.1R for RNA polymerase II second largest subunit (*rpb2*) region (Matheny 2005). PCR protocols for ITS and nrLSU were as described in White et al. (1990), and for *rpb2*, as described in Matheny (2005). PCR products were purified and sequenced by TsingKe Biological Technology Co., Ltd. (Beijing, China).

### Sequence alignment and phylogenetic analyses

Thirty-six sequences (12 for ITS, 12 for nrLSU and 12 for *rpb2*) were newly generated for this study and deposited in GenBank (Table 1). The new sequences were subjected to a BLAST search and relevant related sequences retrieved from GenBank (Table 1).

**Table 1.** DNA sequences used in this study and their voucher specimen number, geographic origin, toxin status, and GenBank accession numbers.

Species	Voucher	Locality	Muscarine	ITS	nrLSU	*rpb2*	References
* Inocybe acriolens *	AU10493	Canada	?	NR_153186	NG_057291	N/A	Type material
	JCS071005D	USA	?	N/A	JN974981	MH577492	Unpublished
* I. albodisca *	PBM1390	USA	–	N/A	EU307819	EU307821	Kropp and Albee-Scott (2010)
* I. alienospora *	PBM3743	Australia	?	KP171104	KM197209	KM245970	Latha and Manimohan (2017)
	REH9667	Australia	?	KP171105	KM197210	KM245971	Unpublished
* I. chelanensis *	PBM491	USA	?	N/A	AY239020	AY337368	Matheny (2005)
	PBM2314	USA	?	N/A	AY239021	AY337369	Matheny (2005)
* I. giacomi *	CLC1321	USA	?	N/A	MK153655	N/A	Unpublished
	JV21543	Sweden	?	N/A	MK153656	N/A	Unpublished
	EL80-12	Sweden	?	N/A	MK153657	N/A	Unpublished
* I. grammata *	PBM2602	USA	–	N/A	JN974977	N/A	Unpublished
	PBM2558	USA	–	N/A	JQ313562	N/A	Unpublished
	2012038	China	–	N/A	KU764690	N/A	Fan and Bau (2017)
* I. hydrocybiformis *	TBGT:12318	India	?	KP171130	KP170911	KM245987	Latha and Manimohan (2017)
	ZT10077	Thailand	?	GQ893016	GQ892971	N/A	Unpublished
* I. lasseroides *	PBM3749	Australia	?	KP171145	KP170924	KM245993	Latha and Manimohan (2017)
	PBM3750	Australia	?	KP171146	KP170925	N/A	Unpublished
* I. papilliformis *	TBGT:10480	India	?	KP171131	KP170912	KM245988	Latha and Manimohan (2017)
	CAL1372	India	?	KY440096	KY549126	N/A	Latha and Manimohan 2017
* I. relicina *	JV10258	Finland	?	N/A	AY038324	AY333778	Matheny (2005)
	EL2-05	Sweden	?	N/A	MN296111	N/A	Unpublished
* I. sierraensis *	DED6101	USA	?	N/A	AY239025	MH249810	Kropp and Matheny (2004)
	DED6477	USA	?	N/A	AY239026	N/A	Kropp and Matheny (2004)
* I. soluta *	EL10706	Sweden	+	N/A	FN550878	N/A	Unpublished
	JV7811F	Finland	+	N/A	JN974987	N/A	Ryberg and Matheny (2012)
* I. sphaerospora *	60-774	Japan	?	AB509953	N/A	N/A	Unpublished
	ZRL20151281	China	?	LT716044	KY418860	KY419006	Unpublished
* I. sphaerospora *	DED8059	Thailand	?	GQ892993	GQ892948	MH577472	Horak et al. (2015)
I. aff. sphaerospora	DED8153	Thailand	?	GQ892994	GQ892949	MH577471	Horak et al. (2015)
	PKSR10	India	?	KJ411954	N/A	KJ411970	Unpublished
*** I. squarrosofulva ***	MHHNU31548 (holotype)	China	+	MZ050799	MW715814	MW574997	This study
	MHHNU31927	China	+	MZ050802	MW715815	MW729766	This study
*** I. squarrosolutea ***	MHHNU8536	China	+	MK250946	MW709445	MW715635	This study
	MHHNU8984	China	+	MK388162	MW709446	MW715636	This study
	MHHNU31006	China	+	MZ050796	MW709457	MW715637	This study
	MHHNU31042	China	+	MZ050800	MW709486	MW715638	This study
	MHHNU31173	China	+	MZ050797	MW715813	MW729760	This study
	MHHNU31427	China	+	MZ050794	MW715804	MW729761	This study
	MHHNU 31434	China	+	MZ050798	MW709488	MW729762	This study
	MHHNU31445	China	+	MZ050801	MW709528	MW729763	This study
	MHHNU31875	China	+	MZ050795	MW709531	MW729764	This study
	MHHNU32151	China	+	MZ050793	MW709532	MW729765	This study
* I. stellatospora *	PRL2716	USA	?	N/A	EU307840	N/A	Kropp and Albee-Scott (2010)
	EL3004	Sweden	?	AM882747	AM882747	N/A	Unpublished
	PBM963	USA	?	N/A	AY038328	AY337403	Matheny (2005)
**Outgroups**							
* Auritella dolichocystis *	Trappe24844	Australia	?	N/A	AY380371	AY337371	Matheny (2005)
	Trappe24843	Australia	?	N/A	AY635764	AY635780	Unpublished
* Inosperma calamistratum *	PBM2351	USA	–	N/A	AY380368	KM245971	Matheny (2005)
	JV11950	USA	–	N/A	EU555452	KM245971	Unpublished
	PBM1105	USA	–	JQ801386	JQ815409	JQ846466	Matheny et al. (2020)
* Mallocybe terrigena *	JV16431	Finland	–	N/A	AY380401	AY333309	Matheny (2005)
	PBM1563	USA	–	N/A	MN178550	N/A	Unpublished
* Nothocybe distincta *	ZT9250	India	?	N/A	EU604546	N/A	Matheny et al. (2020)
* Pseudosperma sororium *	ADW0063	USA	+	JQ408779	JQ319703	JQ421073	Latha and Manimohan (2017)
	PBM3901	USA	+	N/A	MH220278	MH249810	Matheny et al. (2020)
* Tubariomyces inexpectatus *	AH20390	Spain	–	N/A	EU569855	GU907088	Matheny et al. (2020)
* Crepidotus applanatus *	420526MF0534	USA	–	N/A	AF205694	N/A	Kosentka et al. (2013)
	420526MF0689	USA	–	N/A	AY380406	N/A	Matheny et al. (2020)

The new sequences generated in this study are shown in bold. Toxins refer to Kosentka et al. (2013). The “+” indicates the confirmed presence of muscarine, the “?” indicates ambiguous for muscarine, and the “–” indicates a lack of muscarine.

The sequences were aligned using MAFFT v7.310 (Katoh and Standley 2013) and manually edited using BioEdit v7.0.5 (Hall 1999). Maximum likelihood (ML) analysis was performed using RAxML v7.9.1 (Stamatakis 2006) under the GTR + GAMMA + I nucleotide substitution model and performing nonparametric bootstrapping with 1000 replicates. Bayesian inference (BI) was performed in MrBayes v3.2 (Ronquist et al. 2012). The optimal substitution model was determined using the Akaike information criterion (AIC) as implemented in MrModeltest v2.3 (Nylander 2004). The selected substitution model for the three partitions was as follows: General Time Reversible + Gamma (GTR + G) for ITS, and General Time Reversible + Proportion-Invariant + Gamma (GTR + I + G) for nrLSU and *rpb2*. The BI analysis was conducted with the following parameters: four simultaneous Markov chains (**MCMC**), each with two independent runs and trees summarized every 1000 generations. The analyses were completed after 1,000,000 generations when the average standard deviation of split frequencies was 0.009808 for the analysis, and the first 25% generations were discarded as burn-in. The phylograms from ML and BI analyses were visualized with FigTree v1.4.3 (Rambaut 2009).

### Analysis of toxins by ultrahigh-performance liquid chromatography tandem mass spectrometry

The procedure of toxin extraction and detection followed Xu et al. (2020) with slight modifications. A 0.05 g powdery sample of dried mushroom pileus was mixed with 2 mL of a methanol-water solution (7:3 v/v) and vortexed for 30 min at room temperature. The mixture was treated in an ultrasonic bath for 30 min. After centrifugation at 10,000 rpm for 5 min, the supernatant was purified using a QuCHERS–PP column. Subsequently, the extract was mixed with acetonitrile to a final volume of 1.0 mL. The obtained sample solution was centrifuged at 21,000 rpm for 2 min before UPLC–MS/MS analysis. *Lentinulaedodes* was used as a blank sample.

UPLC–MS/MS analysis was carried out with a Waters ACQUITY I-Class UPLC system coupled with a Waters Xevo TQ-S MS/MS system (Waters, Milford, MA, USA). The chromatographic separation was conducted using an ACQUITY UPLC Amide column (2.1 × 100 mm, 1.7 μm; Waters). A gradient elution system used the mobile phase A (acetonitrile) and the mobile phase B (0.05% formic acid aqueous solution) at a flow rate of 0.6 mL/min. The gradient program was as follows: (1) 70–10% A for 1 min, (2) 10% A for 0.5 min, (3) 10–70% A for 0.5 min, and (4) 70% A for 3 min. The analytical column was set to 40 °C, and the injection volume was 2.0 μL. The muscarine content was estimated in the mushroom extract by using standard muscarine (Sigma-Aldrich, St. Louis, MO, USA, Chemical purity ≥ 98%).

A protonated molecular ion ([M + H]^+^ = 174.2) was chosen as the parent ion as well as two daughter ions of 57.0 and 97.0, which were used for qualitative and quantitative detection, respectively. The MS/MS conditions were as follows: ESI^+^ mode; cone, 18 V; collision, 16 V; capillary, 3.0 kV; desolvation temperature, 500 °C; source temperature, 150 °C; desolvation gas flow, 1000 L/Hr; cone gas flow, 150 L/Hr; and collision gas flow, 0.19 mL/min. All the gases were 99.999% pure. Other parameters were used with default values. The product ion confirmation (PIC) was set as follows: scan function; daughter scan; activation threshold level, 500× background noise; minimum activation threshold, 5000 counts; reset threshold level, 50% of act threshold; mass above parent, 100 Da; minimum mass, 50 Da; centroid; scan speed at 5000 amu/s; PIC duration, 0.5 s; and collision energy, 20 V. The analytical results were reported as X ± U (k = 2, *p* = 95%), where X is the analytical content and U is the expanded measurement uncertainty (Eurachem 2012).

## Results

### Phylogenetic data

**Figure 1.**
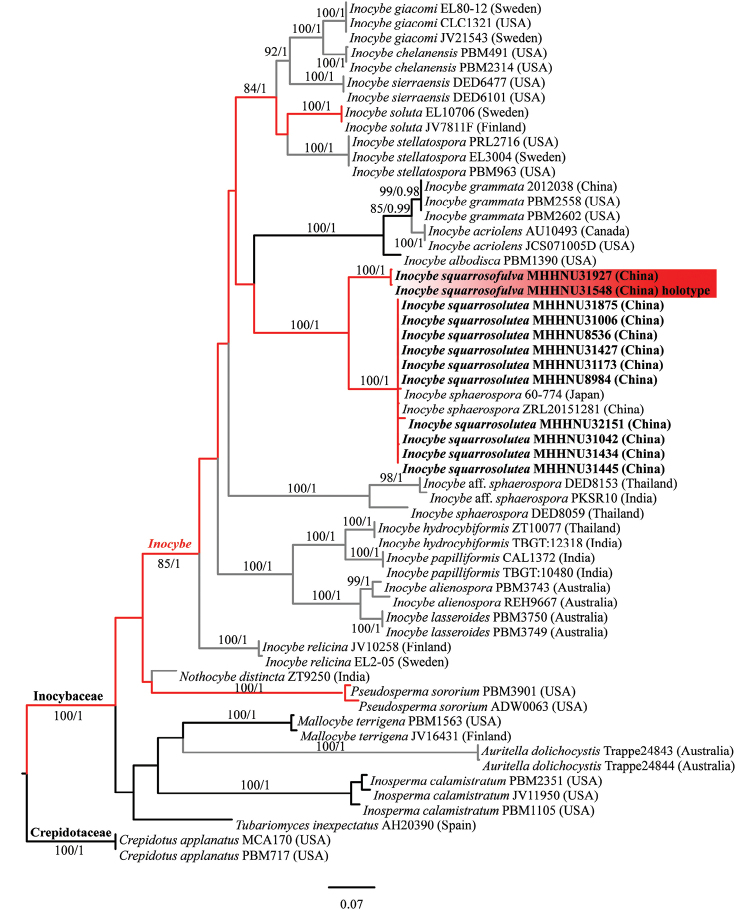
Phylogenetic relationship and placement of *Inocybesquarrosofulva* and *I.squarrosolutea* inferred from the combined dataset (ITS, nrLSU, and *rpb2*) using ML. Bootstrap values ≥80% and Bayesian posterior probabilities ≥0.95 are reported on the branches. Sequences generated in this study are shown in bold. The new species is indicated in red. The red branch indicates the confirmed presence of muscarine, the gray branch indicates ambiguous for muscarine, and the black branch indicates a lack of muscarine.

The combined dataset (ITS, nrLSU, and *rpb2*) contained 1987 total characters and included 58 sequences. The topologies of ML and BI phylogenetic trees obtained in this study were practically the same and the only ML tree with branch lengths and support values is shown in Figure 1. All members of *Inocybes. str.* in the dataset formed a monophyletic lineage with strong support (MLB = 85%, BPP = 1). Ten specimens of *I.squarrosolutea* from China (MHHNU8536, MHHNU8984, MHHNU31006, MHHNU31042, MHHNU31173, MHHNU31427, MHHNU31434, MHHNU31445, MHHNU31875, MHHNU32151) and two samples labeled as “*I.sphaerospora*” from China (ZRL20151281) and Japan (60-774) grouped together in a well-supported lineage (MLB = 100%, BPP = 1.0). The new species, *I.squarrosofulva*, formed a well-supported distinct lineage from *I.squarrosolutea* (MLB = 100%, BPP = 1.0) and is sister to the lineage of *I.squarrosoluta* with significant support (MLB = 100%, BPP = 1.0).

### Taxonomy

#### Inocybe squarrosolutea

Taxon classificationFungiAgaricalesInocybaceae

(Corner & E. Horak) Garrido, Bibliotheca Mycologica 120: 177, 1988.

B9F6591A-BED5-5470-9852-986BE5127D88

Figures 2 , 3 , 6a

≡ Astrosporinasquarrosolutea Corner & E. Horak, Persoonia 10(2): 175, 1979.

##### Basidiomata.

Small to medium-sized. Pileus: 30–60 mm in diameter, bell-shaped to convex when young, and then planar with umbonate center; margin strongly in-rolled or deflexed when young, and then gradually straight when mature; center covered with stout, erect, conic squamules (up to 2 mm high, 1–1.5 mm wide), coarsely fibrillose towards the margin; surface dry, primrose yellow (1A6) to bright yellow (2A5), becoming pale brown (3B6) over the disc. Lamellae crowded (ca. 50–70), 3–5 mm wide, adnexed to adnato-decurrent, often subsinuate; light yellow (1A5) turning to pale yellow-fuscous (2B5), edge concolorous, even. Stipe 35–75 × 4–8 mm, cylindrical or attenuated towards apex, stout, base subbulbous to bulbous, up to 16 mm wide; bright yellow (2A5); apex pruinose, covered with bright yellow(2A5) to orange (2A6), longitudinal, floccose-fibrillose fibrils towards base; dry, solid. Cortina conspicuous present in young specimens. Context pale yellow (1A4) in stipe and cuticle.

##### Basidiospores.

(5.0) 5.5–9.0 (10.0) µm (av. 7.1 μm, SD 1.1 μm) × (4.0) 4.5–6.0 (6.5) µm (av. 5.3 μm, SD 0.6 μm), Q = (1.00) 1.11–1.67 (1.80), Qm = 1.33 ± 0.19 (n = 200 of 10 coll.), nodulose, 6–8 hemispheric knobs, yellow-brown with 5% KOH. Basidia: 17–26 × 7–9 µm, 4-spored, clavate to broadly clavate. Pleurocystidia: 37–67 µm (av. 46.1 μm, SD 3.0 μm) × 10–18 µm (av. 13.4 μm, SD 1.2 μm), Q = 2.80–4.0 (n = 100 of 10 coll.), abundant, broadly fusoid to lageniform; crystalliferous at apex, base usually truncate to obtuse, occasionally tapered into pedicel; metuloid, hyaline, sometimes contain a few small crystals or resinous inclusions, thick-walled, walls up to 1.5 µm thick, bright yellow with 5% KOH. Cheilocystidia similar to pleurocystidia, 35–62 × 9–17 µm; paracystidia: 12–25 × 5–11 µm, abundant, thin-walled, translucent inside, clavate to broadly clavate. Hymenophoral trama: sub-regularly arranged, yellowish with 5% KOH, composed of thin-walled, cylindrical to inflated hyphae 4–23 µm wide. Caulocystidia: 48–98 × 17–22 µm, present at stipe apex, in clusters, similar to those of hymenial cystidia; cauloparacystidia: 20–35 × 10–13 µm, clavate to broadly clavate, thin-walled, nearly hyaline inside, abundant. Pileipellis a trichoderm, regular to subregular, pale brown with 5% KOH, composed of smooth, thin-walled, cylindrical hyphae, 4–8 µm in diameter. Oleiferous hyphae present in pileus and stipe trama, 3–10 µm in diameter, branched. Clamp connections present and common in all tissues.

**Figure 2.**
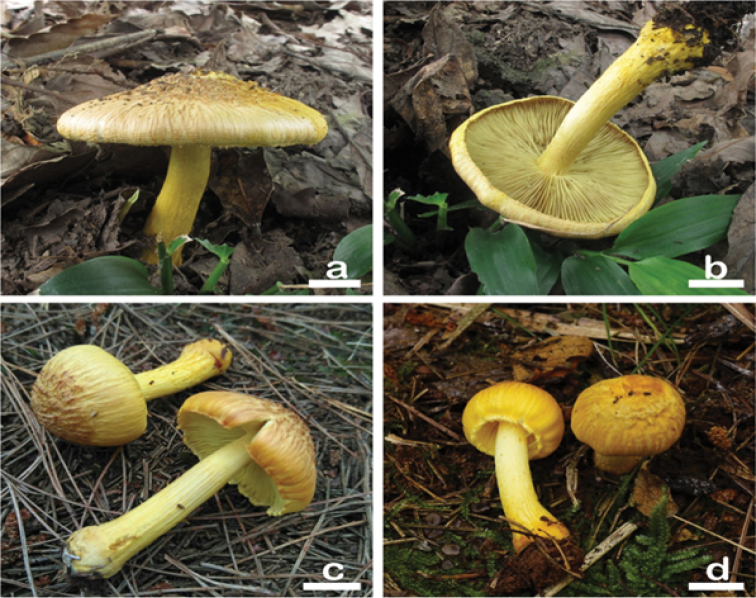
Basidiomata of *Inocybesquarrosolutea***a, b** MHHNU8536 **c** MHHNU31006, and **d** MHHNU31427. Scale bars: 10 mm.

##### Habitat.

Single to scattered in mixed forest dominated by *Pinus* and *Quercus*.

##### Known distribution.

Malaysia (type location) (Horak 1979), China (Hunan Province, Anhui Province).

##### Specimens examined.

China, Hunan Province: Yongshun County, 29 July 2015, MHHNU8536; Yizhang County, 16 September 2016, MHHNU8984; Ningyuan County, 28 May 2017, MHHNU31006; Youxian County, 9 June 2017, MHHNU31042; 18 June 2019, MHHNU31445; Guidong County, 6 July 2018, MHHNU31173; Yongzhou City, 22 May 2019, MHHNU31427; 11 June 2020, MHHNU31875; Qidong County, 2 June 2019, MHHNU31434; Anhui Province, Huangshan City, 11 Aug. 2020, MHHNU32151.

**Figure 3.**
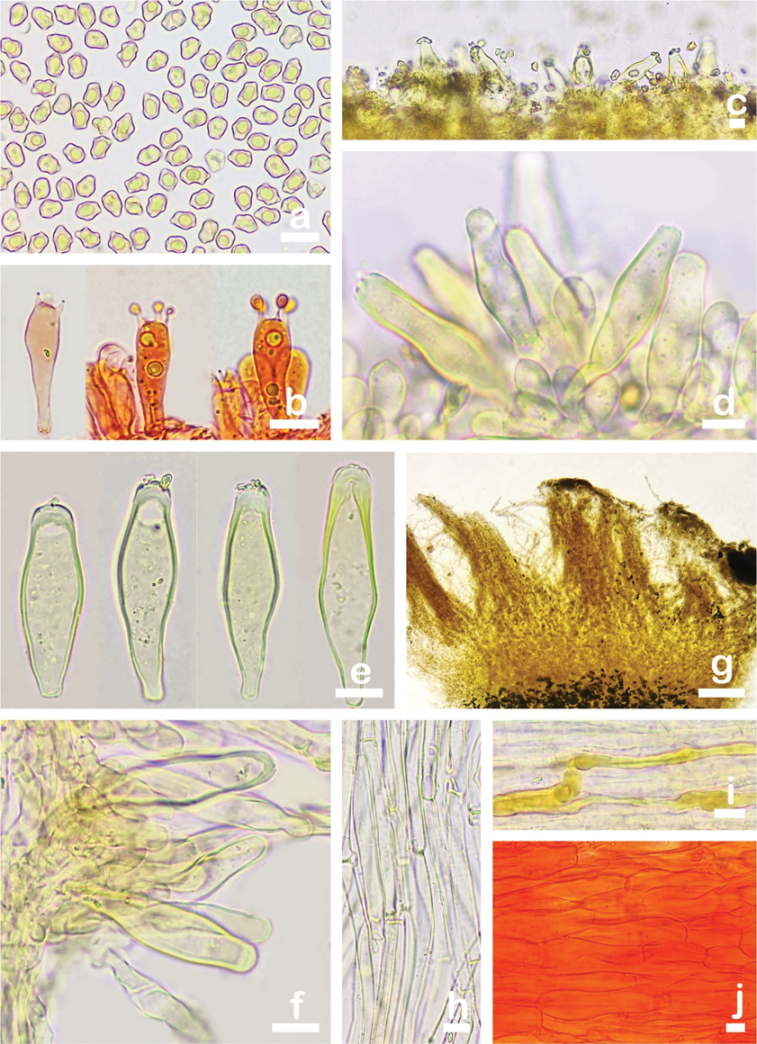
Microscopic features of *Inocybesquarrosolutea* (MHHNU31427) **a** basidiospores **b** basidia with probasidium **c** gill edge **d** cheilocystidia and paracystidia **e** pleurocystidia **f** caulocystidia and cauloparacystidia **g, h** pileipellis **i** oleiferous hyphae, and **j** hymenial hyphae. Scale bars: 10 µm.

#### Inocybe squarrosofulva

Taxon classificationFungiAgaricalesInocybaceae

S.N. Li, Y.G. Fan & Z.H. Chen sp. nov.

F57748B0-05EA-5C02-AB27-0C1B3335B868

839726

Figures 4 , 5 , 6b

##### Etymology.

*Squarrosus* (Latin), squamous; *fulvus* (Latin), brown-orange, referring to its pileus.

##### Holotype.

China. Hunan Province: Zhangjiajie, Badagongshan National Nature Reserve, 29°67.57'N, 109°74.45'E, alt. 1600 m, on ground in subtropical montane forest, 29 July 2019, Z.H. Chen and S.N. Li, MHHNU31548 (GenBank accession no. ITS: MZ050799; nrLSU: MW715814; *rpb2*: MW574997).

##### Diagnosis.

Small to medium-sized basidiomata. Orange-brown to dark brown pileus with squarrose scales. Yellowish brown to brownish , adnexed lamellae. Stipe equal, stout, with distinctly filamentous annulate cortina, pruinose at apex. Odor like raw potatoes. Nodulose basidiospores with six nodules. Hymenial cystidia are broadly fusoid to lageniform, thick-walled. Differs from *Inocybesquarrosolutea* in its orange-brown to dark brown pileus, distinctly filamentous annulus, and less nodulose basidiospores.

**Figure 4.**
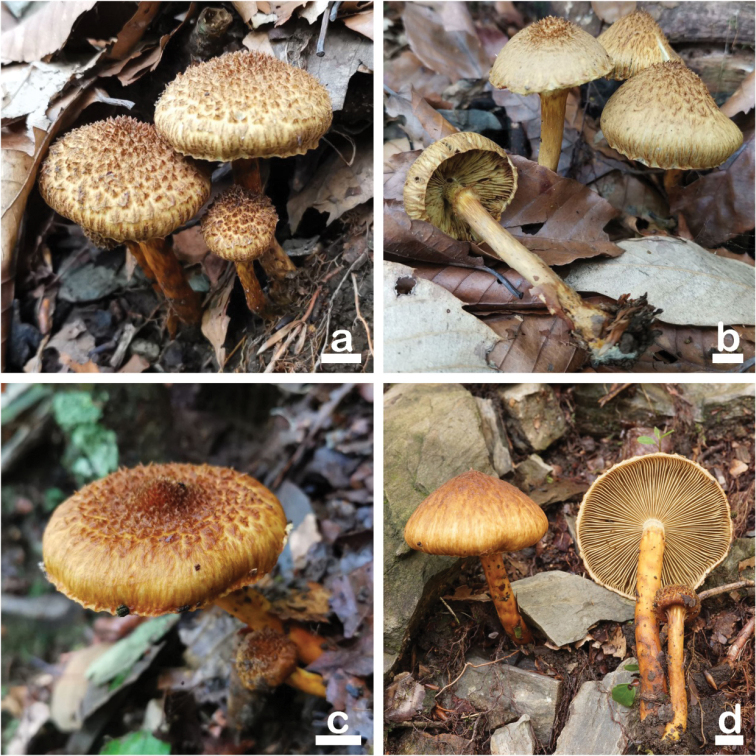
Basidiomata of *Inocybesquarrosofulva***a, b** MHHNU31548 **c, d** MHHNU31927. Scale bars: 10 mm.

##### Basidiomata.

Small to medium-sized. Pileus 25–55 mm in diameter, spherical to bell-shaped when young, and gradually flattened to hemispheric or convex; margin strongly in-rolled when young then decurved or slightly uplifted; yellowish (2A5), center covered with yellow ochre (5C7) to brownish yellow (5C8) erect conical fibrillose scales (up to 1.5 mm high, 1–1.5 mm wide), coarsely fibrillose-rimose towards the margin; pileus with crenellated, nonpersisting fibrillose veil remnants at margin. Lamellae adnexed, crowded (ca. 55–70), up to 4 mm wide; yellowish brown (4C7), becoming brownish (5E4) with age, edge concolorous. Stipe 40–80 × 5–8 mm, cylindrical, equal or slightly enlarged at the base, solid; light yellow (2A3) to yellow ochre (5C7); pruinose with few yellowish-brown (4C7) furfuraceous scales at apex; towards the base covered with numerous, yellow-ochre (5C7), woolly-fibrillose, incomplete zones; dry. Cortina conspicuous, annulate, composed of yellow ochre (5C7) fibrils, and remains at the upper part of the stipe. Context: pale yellow (2A5) in pileus and stipe. Odor like raw potatoes.

**Figure 5.**
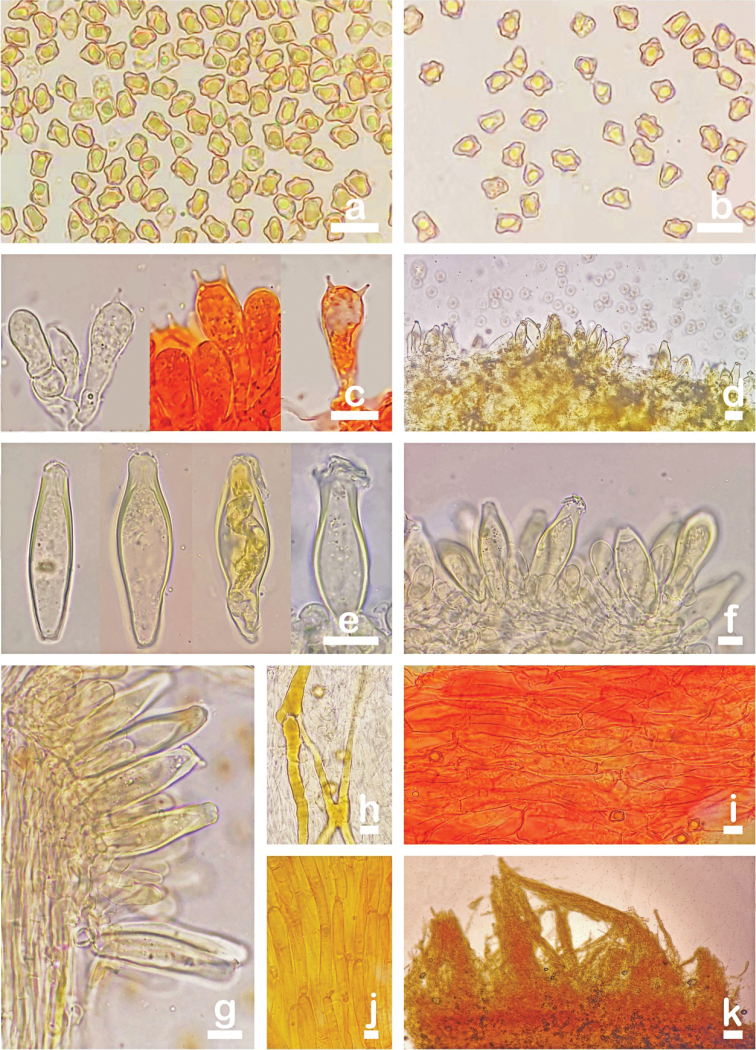
Microscopic features of *Inocybesquarrosofulva* (MHHNU31548, holotype) **a, b** basidiospores, **c** basidia with probasidium **d** gill edge **e** pleurocystidia **f** cheilocystidia and paracystidia **g** caulocystidia and cauloparacystidia **h** oleiferous hyphae **i** hymenial hyphae, and **j, k** pileipellis. Scale bars: 10 µm.

##### Basidiospores.

(4.5) 5.0–7.0 µm (av. 6.6 μm, SD 1.0 μm) × 4.0–6.0 (7.0) (av. 5.3 μm, SD 0.8 μm) µm, Q = (1.00) 1.10–1.67 (1.75), Qm = 1.26 ± 0.16 (n = 80 of 4 coll.), nodulose with six hemispheric knobs, yellowish-brown with 5% KOH, containing a bright yellow oil droplet of uniform size inside. Basidia: 18–24 × 8–10 µm, 4-spored, clavate to broadly clavate. Pleurocystidia: 36–49 µm (av. 43.8 μm, SD 3.9 μm) × 13–18 µm (av. 15.5 μm, SD 2.6 μm), Q = 2.12–3.46 (n = 30 of 2 coll.), mostly hyaline, few with bright yellow oily inclusions, fusiform to broadly fusiform, with crystalliferous apices, obtuse or truncated at base; thick-walled, walls bright yellow with 5% KOH, up to 2 µm thick towards apex. Cheilocystidia: 30–48 × 9–19 µm, similar to pleurocystidia, hyaline. Cheiloparacystidia: 10–23 × 6–12 µm, abundant among cheilocystidia, obovate, elliptic to clavate, thin-walled, hyaline. Hymenophoral trama: regular to subregular, composed of inflated hyphae, up to 18 μm wide, hyaline to lightly yellow with 5% KOH, thin-walled. Pileipellis: a trichoderm, subregular, consisting of cylindrical hyphae 5–13 µm in diameter, walls pale yellow brown with 5% KOH, smooth, thin-walled. Caulocystidia: present at stipe apex, 23–49 × 9–21 μm, in clusters, thick-walled, walls thinner than pleurocystidia, hyaline or with pale yellow intracellular contents. Cauloparacystidia: 8–19 × 3–10 μm, clavate or broadly clavate, hyaline, thin-walled. Oleiferous hyphae present in pileus and stipe trama, 4–11 μm in diameter, branched. Clamp connections seen on all hyphae.

**Figure 6.**
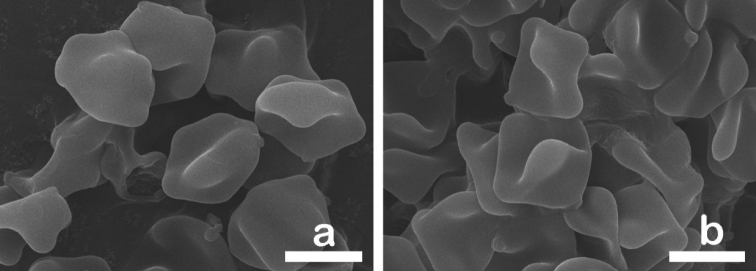
SEM images showing basidiospores of **a***Inocybesquarrosolutea***b***Inocybesquarrosofulva*. Scale bars: 5 µm.

##### Habitat.

On soil in subtropical montane forest dominated by *Faguslucida*.

##### Known distribution.

Known from the type locality.

##### Other examined specimens.

27 July 2020, Z.H. Chen and S.N. Li, MHHNU31927.

### Toxin detection

**Figure 7.**
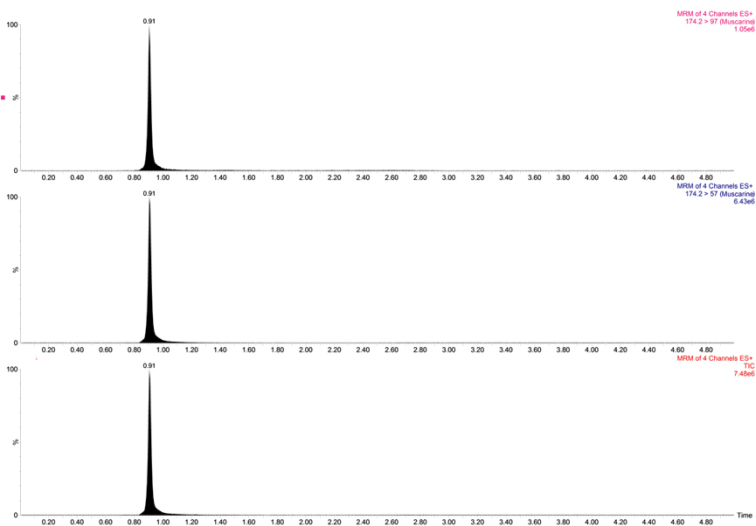
Total ion current (TIC) chromatogram of muscarine in *Inocybesquarrosolutea* (MHHNU31427).

**Figure 8.**
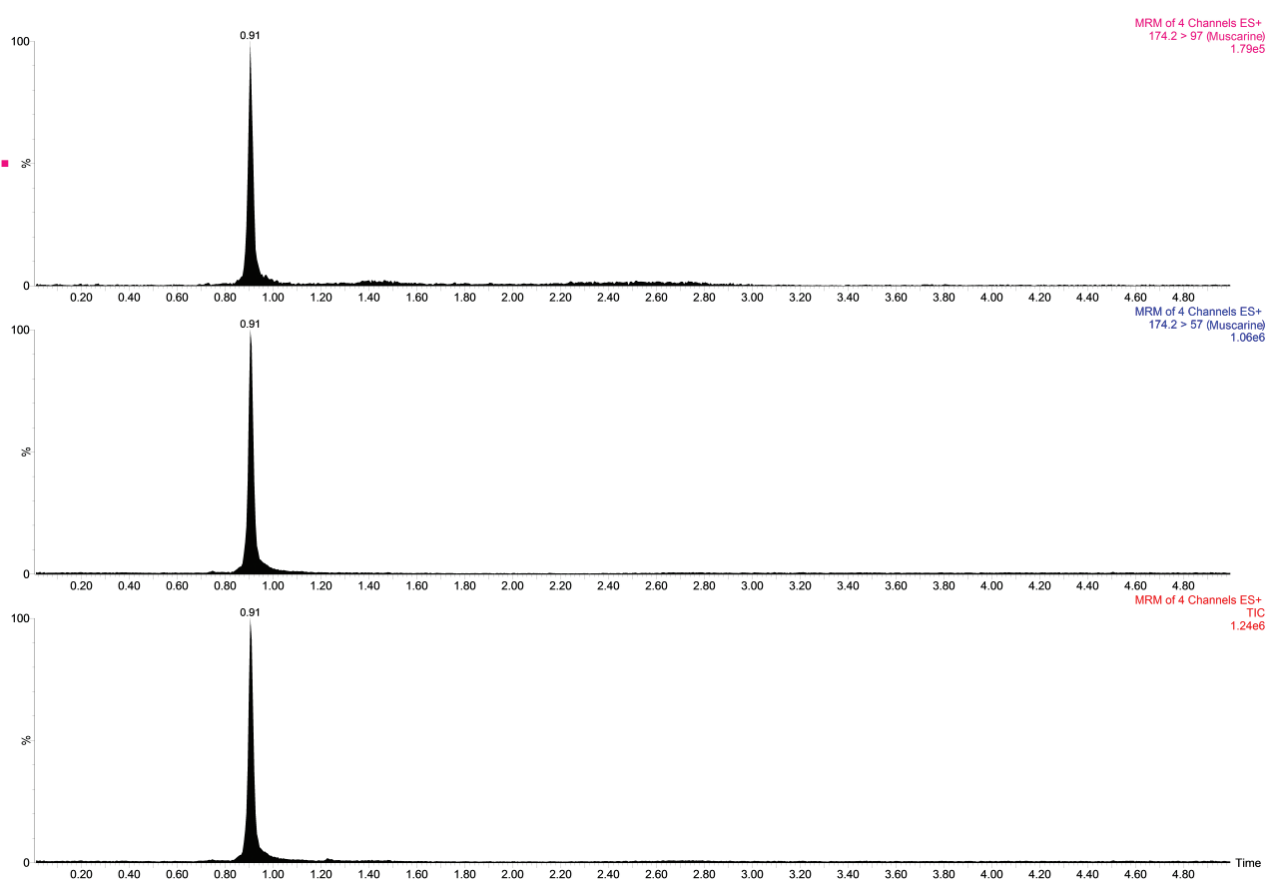
Total ion current (TIC) chromatogram of muscarine in *Inocybesquarrosofulva* (MHHNU31548).

**Figure 9.**
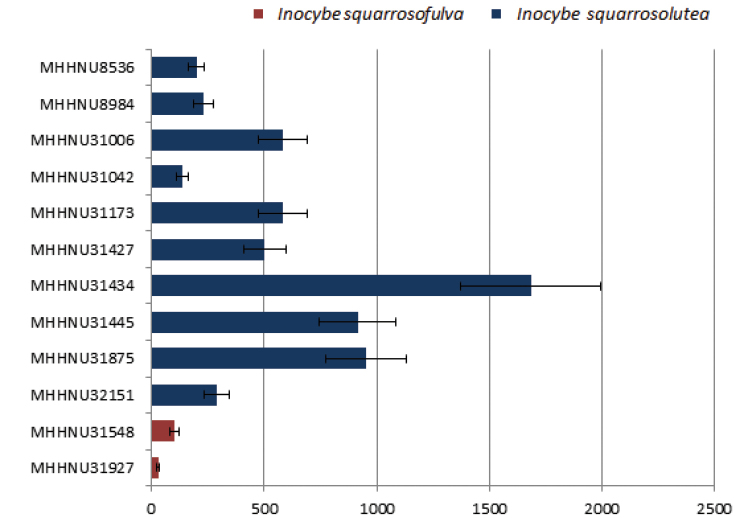
Relative muscarine concentrations measured by UPLC–MS/MS.

Through UPLC–MS/MS detection, we found that both *I.squarrosolutea* and *I.squarrosofulva* contained muscarine (Figs 7, 8). In the qualitative analysis, muscarine was identified by comparing the retention time (0.91 min) and relative deviation (0.6%) within the allowable relative range of 25%. The calibration curve in the matrix blank extract given by Y = 69369X + 6849.33, *R^2^* = 0.9990 (X is injection volume, Y is peak area, and *R^2^* is correlation coefficient) for muscarine concentration was in the range of 0.5–20 ng/mL. The contents of in *I.squarrosolutea* and *I.squarrosofulva* were 136.4 ± 25.4–1683.0 ± 313 mg/kg dry weight and 31.2 ± 5.8–101.8 ± 18.9 mg/kg dry weight, respectively (Fig. 9). Recovery of muscarine ranged from 72.2 to 93.6%; the average recovery was 83.0%.

## Discussion

### Species delimitation

Based on the morphological characteristics, the mushroom was identified as *I.squarrosolutea*, which was first described from Cameron Highlands of Malaysia (Horak 1979). According to the original description, this species is characterized by a large-sized basidiomata, a bright yellow coloration and a scaly pileus and orange fibrillose veil remnants on the stipe. Our Chinese materials fit well with the original description in basidiomata size, outwards appearances, and the shape and size of micro-features. Meanwhile, there are some tiny difference between them. The holotype of *I.squarrosolutea* has longer scales (up to 4 mm) in pileus, smaller basidiospores (4–8 × 5–6 μm), finer basidia (18–26 × 5–7 μm), thicker hymenial cystidia (30–60 × 14–25 μm) (Horak 1979). This species is a close relative of *I.lutea* which, by contrast, has a smaller fruiting body, a smooth pileus, and distinctly smaller basidiospores (Kobayasi 1952; Horak 1979). It is easily for people to confuse *I.squarrosolutea* and *I.sphaerospora* because of their similar appearance. In fact, they can be easily distinguished by their basidiospores. The basidiospores of *I.squarrosolutea* are nodulose, while those of *I.sphaerospora* are globose (Kobayasi 1952; Horak et al. 2015). In phylogenetic analysis (Fig. 1) the specimens of *I.sphaerospora* identified by Horak et al. (2015) formed a monophyletic lineage with strong support (MLB = 100%, BPP = 1), and was distinct from *I.squarrosolutea*. However, the two materials labeled as *I.sphaerospora* from China (ZRL20151281) and Japan (60-774), cluster together with *I.squarrosolutea* in the phylogenetic tree, indicating an inaccurate identification of these two materials.

*Inocybesquarrosofulva* is characterized by its orange brown to dark brown pileus with squarrose scales, distinctly filamentous annulate cortina in stipe, stipe pruinose only near the apex, nodulose basidiospores with six hemispheric knobs, and its odor like raw potatoes. Phylogenetic analyses revealed that *I.squarrosofulva* is an independent lineage in *Inocybes. str.* and is sister to *I.squarrosolutea*. However, *I.squarrosolutea* differs in having primrose yellow to bright yellow pileus with less squarrose scales, no distinctly filamentous annulus cortina in the stipe, a subbulbous to bulbous stipe base, a less nodulose basidiospores, and smaller hymenial cystidia. Microscopically, *I.lutea* is similar to new species in shape and size of pleurocystidia and basidiospores, but the pileus of *I.lutea* covered with radially fibrils and pruinate all over the stipe (Kobayasi 1952; Horak 1979). Lastly, a Papua New Guinea material described as *Inocybeluteifolia* (E. Horak) Garrido 1988 (non *Inocybeluteifolia* A.H. Sm. 1941), which is an illegitimate species name, resembles the new species in macromorphology, but it has smaller basidiomata, larger cheilocystidia and pleurocystidia (55–85 × 10–20 μm), no caulocystidia on the stipe, and a fish-like odor (Horak 1979).

Kuyper (1986) recognized two groups on the (informal) level of “supersection”, viz. *Cortinatae* and *Marginatae*, according to the different development mode and, hence, absence or presence of a cortina, and the nature of stipe covering. Due to their presence of a cortina and pruinose at the apex of the stipe, both *I.squarrosolutea* and *I.squarrosofulva* might be classified in supersection Cortinatae. The morphological characteristics corresponding to the phylogenetic branches are not yet clear (Matheny 2005; Matheny et al. 2020), so the infrageneric framework of *Inocybes. str.* is still unknown and its characterization requires more research.

### Toxicity in *Inocybe*

According to the literature, muscarine was first isolated and identified from *Amanitamuscaria*, but the actual muscarine content of *A.muscaria* is very low (usually around 0.0003% of the fresh weight) (Waser 1961). Conversely, muscarine concentrations are much higher in *Inocybes. l.* spp. (Malone et al. 1962). Brown et al. (1962) detected the muscarine contents of 34 species of *Inocybes. l.* by paper chromatographic method, ranging from 0.01 to 0.80% in approximately 75% of them. Kosentka et al. (2013) used liquid chromatography–tandem mass spectrometry (LC–MS/MS) to determine whether muscarine was present in 30 new samples of *Inocybes. l.* Of the 30 species they assayed, eleven species tested positive for presence of muscarine, ranging from ca. 0.00006% to 0.5%. Xu et al. (2020) determined the muscarine content of *I.serotina* by UPLC-MS/MS, and its muscarine content was 324.0 ± 62.4 mg/kg. In our study, the toxin content in each sample was determined using a linear regression equation according to the peak area of the UPLC–MS/MS analysis chromatogram of the test sample (Figs 7, 8). The results showed that both species contained muscarine; the content of muscarine in *I.squarrosolutea* ranged from 136.4 ± 25.4 to 1683.0 ± 313 mg/kg dry weight and the content in *I.squarrosofulva* was generally lower, ranging from 31.2 ± 5.8 to 101.8 ± 18.9 mg/kg dry weight (Fig. 9). Calculated on a dry-weight basis, the percentage concentrations were 0.01–0.17% for *I.squarrosolutea* and 0.003–0.01% for *I.squarrosofulva*, which is in range of previous reports.

There are some differences in the muscarine content of different poisonous *Inocybe* spp., even within a particular species. The capacity of *Inocybe* species to accumulate muscarine may be influenced by certain hereditary (infraspecific races) or environmental factors (Brown et al. 1962). In this study, the differences in muscarine content among specimens of *I.squarrosolutea* may be related to region and climate. *I.squarrosofulva* MHNNU31548 and *I.squarrosofulva* MHNNU31927 were collected in the same place in different years. The weather was sunny at the time of the collection of *I.squarrosofulva* MHNNU31548, and there was heavy rain at the time of the collection of *I.squarrosofulva* MHNNU31927, so it is presumed that the difference in muscarine content may be related to rainwater washing.

## Supplementary Material

XML Treatment for Inocybe squarrosolutea

XML Treatment for Inocybe squarrosofulva
